# *In vitro *antibacterial activity of some plant essential oils

**DOI:** 10.1186/1472-6882-6-39

**Published:** 2006-11-30

**Authors:** Seenivasan Prabuseenivasan, Manickkam Jayakumar, Savarimuthu Ignacimuthu

**Affiliations:** 1 Entomology Research Institute, Loyola College, Chennai – 600 034, India.

## Abstract

**Background:**

To evaluate the antibacterial activity of 21 plant essential oils against six bacterial species.

**Methods:**

The selected essential oils were screened against four gram-negative bacteria (*Escherichia coli*, *Klebsiella pneumoniae*, *Pseudomonas aeruginosa*, *Proteus vulgaris*) and two gram-positive bacteria *Bacillus subtilis *and *Staphylococcus aureus *at four different concentrations (1:1, 1:5, 1:10 and 1:20) using disc diffusion method. The MIC of the active essential oils were tested using two fold agar dilution method at concentrations ranging from 0.2 to 25.6 mg/ml.

**Results:**

Out of 21 essential oils tested, 19 oils showed antibacterial activity against one or more strains. Cinnamon, clove, geranium, lemon, lime, orange and rosemary oils exhibited significant inhibitory effect. Cinnamon oil showed promising inhibitory activity even at low concentration, whereas aniseed, eucalyptus and camphor oils were least active against the tested bacteria. In general, *B. subtilis *was the most susceptible. On the other hand, *K. pneumoniae *exhibited low degree of sensitivity.

**Conclusion:**

Majority of the oils showed antibacterial activity against the tested strains. However Cinnamon, clove and lime oils were found to be inhibiting both gram-positive and gram-negative bacteria. Cinnamon oil can be a good source of antibacterial agents.

## Background

The spread of drug resistant pathogens is one of the most serious threats to successful treatment of microbial diseases. Down the ages essential oils and other extracts of plants have evoked interest as sources of natural products. They have been screened for their potential uses as alternative remedies for the treatment of many infectious diseases [1]. World Health Organization (WHO) noted that majority of the world's population depends on traditional medicine for primary healthcare. Medicinal and aromatic plants which are widely used as medicine and constitute a major source of natural organic compounds.

Essential oils have been shown to possess antibacterial, antifungal, antiviral insecticidal and antioxidant properties [2,3]. Some oils have been used in cancer treatment [4]. Some other oils have been used in food preservation [5], aromatherapy [6] and fragrance industries [7]. Essential oils are a rich source of biologically active compounds. There has been an increased interest in looking at antimicrobial properties of extracts from aromatic plants particularly essential oils [8]. Therefore, it is reasonable to expect a variety of plant compounds in these oils with specific as well as general antimicrobial activity and antibiotic potential [9].

Essential oils (also called volatile oils) are aromatic oily liquids obtained from plant materials (flowers, buds, seeds, leaves, twigs, bark, herbs, wood, fruits and roots). They can be obtained by expression, fermentation or extraction but the method of steam distillation is most commonly used for commercial production. An estimated 3000 essential oils are known, of which 300 are commercially important in fragrance market [7]. Essential oils are complex mixers comprising many single compounds. Chemically they are derived from terpenes and their oxygenated compounds. Each of these constituents contributes to the beneficial or adverse effects.

Essential oils such as aniseed, calamus, camphor, cedarwood, cinnamon, citronella, clove, eucalyptus, geranium, lavender, lemon, lemongrass, lime, mint, nutmeg, orange, palmarosa, rosemary, basil, vetiver and wintergreen have been traditionally used by people for various purposes in different parts of the world (Table 1). Cinnamon, clove and rosemary oils had shown antibacterial and antifungal activity [10]; cinnamon oil also possesses antidiabetic property [11]. Anti-inflammatory activity has been found in basil [12]. Lemon and rosemary oils possess antioxidant property [13,14]. Peppermint and orange oils have shown anticancer activity [15,16]. Citronella oil has shown inhibitory effect on biodegrading and storage-contaminating fungi [17]. Lime oil has shown immunomodulatory effect in humans [16]. Lavender oil has shown antibacterial and antifungal activity; it was also found to be effective to treat burns and insect bites [18].

**Table 1:** List of selected essential oils and their properties.

**Common name**	**Botanical name (Family)**	**Properties **[37, 42, 43]
Aniseed oil	*Pimpinella anisum *(Umbelliferae)	Carminative, stimulant, expectorant, condiment and flavouring agent.
Calamus oil	*Acorus calamus *(Araceae)	Carminative, bitter stimulant, vermifuge and insect repellent
Camphor oil	*Cinnamomuum camphora *(Lauraceae)	Rubefacient, tooth powder and cosmetic agent.
Cedarwood oil	*Cedrus atlantica *(Coniferae)	Antiseptic, astringent, diuretic, fungicidal, sedative and stimulant.
Cinnamon oil	*Cinnamomum zeylanicum *(Lauraceae)	Carminative, stomachic, astringent, stimulant and antiseptic.
Citronella oil	*Cymbopogon nardus *(Gramineae)	Perfumery, mosquito repellent and flavouring agent.
Clove oil	*Eugenia caryophyllus *(Myrtaceae)	Dental analgesic, carminative, stimulant and antiseptic.
Eucalyptus oil	*Eucalyptus globulu*s (Myrtaceae)	Counter-irritant, antiseptic, expectorant, cough reliever.
Geranium oil	*Pelargonium graveolens *(Geraniaceae)	Flavouring agent and stimulant.
Lavender oil	*Lavandula angustifolia *(Labiatae)	Stimulant and flavouring agent.
Lemon oil	*Citrus limon *(Rutaceae)	Carminative, stimulant, perfuming and flavouring agent.
Lemongrass oil	*Cymbopogon citratus *(Graminae)	Flavouring agent, antiseptic and deodorant.
Lime oil	*Citrus aurantium *(Rutaceae)	Stomachic, carminative and flavouring agent.
Nutmeg oil	*Myristica fragrans *(Myristicaceae)	Stimulant, anti rheumatic and carminative.
Orange oil	*Citrus sinensis *(Rutaceae)	Stomachic, carminative and flavouring agent.
Palmarosa oil	*Cymbopogon martini *(Graminae)	Cosmetic, anti rheumatism and insect repellent.
Peppermint oil	*Mentha piperita *(Labiatae)	Digestent, stimulant and tonic.
Rosemary oil	*Rosmarinus officinalis *(Labiatae)	Carminative, stimulant and flavouring agent.
Basil oil	*Ocimum sanctum *(Labiatae)	Antibacterial, insecticidal, stimulant, stomachic and diaphoretic.
Vetiver oil	*Vetiveria zizanioides *(Graminae)	Stimulant, refrigerant, flavouring agent, stomachic and fixative.
Wintergreen oil	*Gaultheria fragrantissima *(Ericaceae)	Irritant, vermicide agent and flavouring agent.

In spite of all the information available on the 21 oils selected for this study, we were not able to find antibacterial activity for all those oils. Hence this study was undertaken with the intention of finding out the efficacy of these essential oils as antimicrobial agents for commercial purposes.

## Methods

### Essential oils

Twenty-one essential oils obtained from Tegraj & Co (P) Ltd, India (commercial producers of plant essential oils and aromatic substances) were used in this study (Table 1). These oils were selected based on literature survey and their use in traditional medicine. Quality of the oils was ascertained to be more than 98% pure.

### Test organism

Microorganisms were obtained from the Department of Microbiology, Christian Medical College, Vellore, India and Institute of Basic Medical Sciences (IBMS), Chennai, India. Four strains of gram-negative bacteria [*Escherichia coli *(ATCC 25922), *Klebsiella pneumoniae *(ATCC 15380), *Pseudomonas aeruginosa *(ATCC 27853), *Proteus vulgaris *(MTCC 1771)] and two strains of gram-positive bacteria [*Bacillus subtilis *(MTCC 441) and *Staphylococcus aureus *(ATCC 25923)] were used. The cultures of bacteria were maintained in their appropriate agar slants at 4°C throughout the study and used as stock cultures.

### Antibacterial assay

Screening of essential oils for antibacterial activity was done by the disk diffusion method, which is normally used as a preliminary check and to select between efficient essential oils [2]. It was performed using an 18 h culture at 37°C in 10 ml of Mueller Hinton Broth. The cultures were adjusted to approximately 10^5^CFU/ml with sterile saline solution. Five hundred microliters of the suspensions were spread over the plates containing Mueller-Hinton agar using a sterile cotton swab in order to get a uniform microbial growth on both control and test plates. The essential oils were dissolved in 10% aqueous dimethylsulfoxide (DMSO) with Tween 80 (0.5% v/v for easy diffusion) and sterilized by filtration through a 0.45 μm membrane filter. Under aseptic conditions, empty sterilized discs (Whatman no. 5, 6 mm dia) were impregnated with 50 μL of different concentrations (1:1, 1:5, 1:10, 1:20) of the respective essential oils and placed on the agar surface [19]. Paper disc moistened with aqueous DMSO was placed on the seeded petriplate as a vehicle control. A standard disc containing streptomycin (25 μg/disc) was used as reference control. All petridishes were sealed with sterile laboratory parafilm to avoid eventual evaporation of the test samples. The plates were left for 30 min at room temperature to allow the diffusion of oil, and then they were incubated at 37°C for 18 h (18 h was fixed as the optimum since there was no change in the inhibition up to 24 h) After the incubation period, the zone of inhibition was measured with a calliper. Studies were performed in triplicate, and mean value was calculated. The means were analysed by one way analysis of variance (ANOVA) followed by Tukey's post hoc multiple comparison test using SPSS software package version 13.0 for windows. The results were expressed as mean ± SD. *P *values <0.05 were considered as significant.

### MIC assay

Based on the previous screening seven essential oils (Cinnamon, clove, geranium, lemon, lime, orange and rosemary oils) were identified to have potent antibacterial activity and their Minimum Inhibitory Concentrations (MIC) were determined. The agar dilution method recommended by the National Committee for Clinical Laboratory Standards [20] was used with the following modification; a final concentration of 0.5% (v/v) Tween-20 (Sigma) was incorporated into the agar medium to enhance oil solubility. A series of two fold dilution of each oil, ranging from 0.2 to 25.6 mg/ml, was prepared in Muellur Hinton agar at 48°C. Plates were dried at room temperature for 30 min prior to spot inoculation with 3 μl aliquots of culture containing approximately 10^5 ^CFU/ml of each organism. Inoculated plates were incubated at 37°C for 18 h and the MIC was determined. Experiments were carried out in triplicate. Inhibition of bacterial growth in the plates containing test oil was judged by comparison with growth in blank control plates. The MICs were determined as the lowest concentration of oil inhibiting visible growth of each organism on the agar plate [21].

### Gas chromatography mass spectrometry (GC/MS)

The most potent oil, (cinnamon oil) was analysed using GC/MS (Shimadzu capillary GC-quadrupole MS system QP 5000) with two fused silica capillary column DB-5 (30 μm, 0.25 mm i.d, film thickness 0.25 μm) and a flame ionization detector (FID) which was operated in EI mode at 70 eV. Injector and detector temperatures were set at 220°C and 250°C, respectively. One microliter essential oil solution in hexane was injected and analyzed with the column held initially at 60°C for 2 min and then increased by 3°C/min up to 300°C. Helium was employed as carrier gas (1 ml/min). The relative amount of individual components of the total oil is expressed as percentage peak area relative to total peak area. Qualitative identification of the different constituents was performed by comparison of their relative retention times and mass spectra with those of authentic reference compounds, or by retention indices (RI) and mass spectra.

## Results

The anti-bacterial activity of twenty-one selected essential oils against six bacterial species is summarized in Table 2 and 3. The results revealed that the selected essential oils showed antibacterial activity with varying magnitudes. The zone of inhibition above 7 mm in diameter was taken as positive result. Generally most of the tested organisms were sensitive to many of the essential oils. Out of 21 essential oils tested, 19 showed antibacterial activity against one or more bacteria. Cinnamon oil, lime oil, geranium oil, rosemary oil, orange oil, lemon oil and clove oil showed maximum activity against all the bacterial species tested. On the other hand, aniseed oil, eucalyptus oil and camphor oil failed to inhibit any of the tested strains. Both gram-positive and gram-negative bacteria were sensitive to the potent essential oils. *P. aeruginosa *and *P. vulgaris *were inhibited by 19 oils, followed by *B. subtilis *(18 oils), *S. aureus *(14 oils), *E. coli *(12 oils) *and K. pneumoniae *(9 oils). In general cinnamon oil showed significant inhibitory effect against *P. aeruginosa *(33.3 mm), *B. subtilis *(29.9 mm), *P. vulgaris *(29.4 mm), *K. pneumoniae *(27.5 mm) and *S. aureus *(20.8 mm). Moderate effects were seen in lime oil, clove oil and lemon oil. No obvious difference in susceptibility was found between gram-negative and gram-positive bacteria. There was no inhibition of growth with the vehicle control (10% DMSO).

**Table 2:** Antimicrobial activity of 21 essential oils against *S. aureus, B. subtilis *and *K. pneumoniae *using disc diffusion method

**Oil Name**	***S. aureus***				***B. subtilis***				***K. pneumoniae***			
Concentration	1:1	1:5	1:10	1:20	1:1	1:5	1:10	1:20	1:1	1:5	1:10	1:20

Aniseed oil	-	-	-	-	12.1 ± 1.0^klm^	10.8 ± 0.2^ij^	-	-	-	-	-	-
Calamus oil	-	-	-	-	17.9 ± 0.9^def^	14.7 ± 0.5^cd^	12.6 ± 0.5 ^c^	11.3 ± 1.2^d^	-	-	-	-
Camphor oil	-	-	-	-	-	-	-	-	8.5 ± 0.5^e^	-	-	-
Cedar wood	-	-	-	-	13.4 ± 0.5^jkl^	12.2 ± 0.5^ghi^	99 ± 0^fg^	-	-	-	-	-
Cinnamon oil	20.8 ± 0.5 ^a^	18.7 ± 0.2^a^	14.8 ± 0.2^b^	13.7 ± 0.28^b^	29.9 ± 0.7 ^a^	27.8 ± 1.1 ^a^	24.1 ± 1.1 ^a^	22.8 ± 0.2^b^	27.5 ± 0.5^a^	23.5 ± 0.5^a^	20.9 ± 0.2^a^	18.6 ± 0.5^a^
Citronella oil	15.8 ± 0.3^bc^	10.3 ± 0.5^de^	-	-	14.6 ± 0.5^ij^	12.5 ± 0.5^ghi^	10.3 ± 0.5^de^	9.3 ± 0.5^ef^	-	-	-	-
Clove oil	16.3 ± 0.5 ^b^	14.0 ± 0^bc^	8.1 ± 1.1^d^	9.8 ± 0.57^c^	14.5 ± 0.2^hij^	13.1 ± 0.2^ef^	10.1 ± 1.0^def^	8.9 ± 0.2^f^	16.2 ± 0.5^c^	14.4 ± 0.5^b^	8.4 ± 0.5^c^	-
Eucalyptus oil	-	-	-	-	-	-	-	-	-	-	-	-
Geranium oil	10.4 ± 0.5^e^	8.9 ± 0.2^e^	-	-	17.2 ± 0.2^defg^	14.7 ± 0.5^cde^	-	-	10.8 ± 0.2^d^	9 ± 0^c^	-	-
Lavender	-	-	-	-	12.5 ± 0.4^jk^	11.1 ± 0.2^hi^	-	-	-	-	-	-
Lemon oil	17.5 ± 1.1^b^	14.5 ± 0.2^b^	8.3 ± 0.2^d^	-	16.9 ± 0.2^efgh^	14.5 ± 0.5^cdef^	8.9 ± 0.5 ^g^	-	16.5 ± 0.5^c^	15.3 ± 1.1^b^	8.7 ± 0.5^c^	-
Lemongrass oil	11.4 ± 1.0 ^de^	8.9 ± 0.2^e^	-	-	16.7 ± 0.5^fghi^	15.1 ± 0.7 ^c^	8.9 ± 1.0^fg^	-	8.3 ± 0.5^e^	-	-	-
Lime oil	14.2 ± 0.5^cd^	12.7 ± .2^bcd^	10.3 ± 0.	9 ± 0.00^c^	23.9 ± 1.1 ^c^	20.8 ± 0.7 ^b^	15.8 ± 0.2 ^b^	14.1 ± 1.1^c^	16.1 ± 1.1^c^	14.6 ± 0.5^b^	12.9 ± 0.5^b^	12.6 ± 0.5^b^
Nutmeg	11.7 ± 0.8^de^	9.1 ± 0.2^e^	-	-	11.5 ± 0.5^mn^	10.9 ± 0.2^hij^	-	-	-	-	-	-
Orange oil	12.8 ± 0.2^cd^	10.6 ± 0.2^cde^	-	-	18.6 ± 0.8^de^	15.7 ± 1.2 c	9.4 ± 0.5^efg^	10.6 ± .5^e^	11.9 ± 0.7^d^	9.31.1^c^	-	-
Palmarosa oil	12.5 ± 0.2^cd^	10.9 ± 0.5^de^	8 ± 0^d^	-	15.4 ± 0.5^ghi^	14.2 ± 1^cdef^	12.5 ± 0.4 ^c^	11.3 ± 0.5^d^	-	-	-	-
Peppermint oil	8.1 ± 0.5^f^	-	-	-	10.6 ± 0.5^mn^	8.6 ± 0.4^k^	-	-	-	-	-	-
Rosemary oil	12.5 ± 1^d^	10.6 ± 1.0^e^	8.6 ± 0.2^d^	-	14.7 ± 1^hi^	12.8 ± 0.5^efg^	11.2 ± 0.5^cd^	8.9 ± 0.2^f^	11.9 ± 0.5^d^	9.9 ± 0.7^c^	-	-
Basil oil	10.3 ± 0.5^e^	8.3 ± 0.5^e^	-	-	-	-	-	-	-	-	-	-
Vetiver oil	-	-	-	-	18.9 ± 0.9 ^d^	15.6 ± 0.5^c^	12.8 ± 0.2 ^c^	11.6 ± 0.7^d^	-	-	-	-
Wintergreen	8.7 ± 0.51^f^	-	-	-	9.9 ± 0.5 ^n^	9.1 ± 0.1^jk^	-	-	-	-	-	-
Streptomycin**	20.9 ± 0.5				26.9 ± 0.5				20.9 ± 0.9			
Vehicle control	-				-				-			

** Streptomycin disc (25 μg) as a positive reference standard; Values are mean inhibition zone (mm) ± S.D of three replicates

Means within a column followed by different letters are statistically significant by tukey's test at p = 0.05; - no activity.

**Table 3:** Antimicrobial activity of 21 essential oils against *P. vulgaris, P. aeruginosa *and *E. coli *using disc diffusion method

**Oil Name**	***P. vulgaris***				***P. aeruginosa***				***E. coli***			
Concentration	1:1	1:5	1:10	1:20	1:1	1:5	1:10	1:20	1:1	1:5	1:10	1:20

Aniseed oil	-	-	-	-	-	-	-	-	-	-	-	-
Calamus oil	11.1 ± 0.7^gh^	9.2 ± 0.5^h^	-	-	13.8 ± 0.5^ef^	12.8 ± 0.4^f^	9.2 ± 0.7 ^d^	8.3 ± 0.5 ^d^	-	-	-	-
Camphor oil	8.8 ± 0.7 ^hi^	-	-	-	10 ± 0.5^gh^	-	-	-	8.8 ± 1^d^	-	-	-
Cedar wood	14.1 ± 1.1^f^	12.9 ± 0.5^g^	8.2 ± 0.5^f^	-	15.6 ± 0.5^de^	14.1 ± 0.9^ef^	8.23 ± 0.5^d^	-	-	-	-	-
Cinnamon oil	29.4 ± 0.5^a^	27 ± 0^a^	18.6 ± 1.52^a^	14.1 ± 0.4^b^	33.3 ± 1.6^a^	32.2 ± 0.5^a^	24.4 ± 0.5^a^	21 ± 0^a^	29.8 ± 0.7^a^	26 ± 1^a^	23.8 ± 1^a^	21 ± 0.2^a^
Citronella oil	19.5 ± 0.5^cd^	11.3 ± 0.7^g^	-	-	12.1 ± 0.5^fg^	10.4 ± 0.5^g^	-	-	-	-	-	-
Clove oil	20.1 ± 1^c^	18.2 ± 0.5^d^	11.8 ± 0.5^d^	8 ± 0^e^	17.4 ± 0.7^d^	15.5 ± 0.2^e^	8.5 ± 0.5^d^	-	17.4 ± 2^c^	15.1 ± 0.5^c^	10.8 ± 0.5^e^	8 ± 0^c^
Eucalyptus oil	-	-	-	-	-	-	-	-	8.5 ± 1^d^	-	-	-
Geranium oil	19 ± 0.2^de^	14.6 ± 0.2^e^	11 ± 1.5^ef^	8.3 ± 0^e^	21.5 ± 0.2^c^	19.2 ± 1.2^d^	9.4 ± 0.5^d^	-	14.1 ± 1^c^	10.4 ± 1^d^	-	-
Lavender	10.8 ± 0.5^ghi^	9.3 ± 0.5^h^	-	-	12.1 ± 0.9^fg^	9.9 ± 0.7^g^	-	-	-	-	-	-
Lemon oil	22.5 ± 0.5^b^	18.9 ± 0.5^d^	15.5 ± 0.5^c^	10.7 ± 0.2^d^	21.4 ± 1.1^c^	19.9 ± 0.7^cd^	9.1 ± 0.2^d^	-	21.6 ± 0.5^b^	18.9 ± 0.5^b^	10.6 ± 0.2^e^	-
Lemongrass oil	14.6 ± 0.7^f^	12.1 ± 0.2^g^	9.5 ± 0.5^ef^	-	23.4 ± 1^c^	19.6 ± 0.5^d^	9.1 ± 0.5^d^	-	-	-	-	-
Lime oil	27.8 ± 0.5^a^	24 ± 0^b^	17.9 ± 0.1^ab^	13 ± 1^c^	25.5 ± 1^b^	23.5 ± 0.5^b^	13.6 ± 0.7^c^	9.3 ± 0.8^c^	22.7 ± 0.5^b^	18.9 ± 1^b^	15.6 ± 0.5^c^	17.6 ± 1.5^b^
Nutmeg	14.5 ± 0.7^f^	11.9 ± 0.2^g^	8 ± 0 ^f^	-	15.5 ± 0.5^de^	12.9 ± 1^f^	-	-	-	-	-	-
Orange oil	23.7 ± 0.7^b^	22.2 ± 0.7 ^c^	16.5 ± 0.2^bc^	10.7 ± 0.5^d^	21.6 ± 0.2^c^	18.8 ± 0.2^d^	9.4 ± 0.5^d^	-	22.9 ± 0.5^b^	19.4 ± 0.7^b^	12.4 ± 0.2^d^	-
Palmarosa oil	19 ± 0^cd^	15.8 ± 0.2 ^e^	9.9 ± 0.2 ^def^	-	14.4 ± 0.5^ef^	13.2 ± 0.5^ef^	9.2 ± 0.2^d^	-	8.2 ± 0.5^d^	-	-	-
Peppermint oil	8.5 ± 0.2^i^	-	-	-	13.4 ± .52^ef^	11.2 ± 0.8^g^	-	-	-	-	-	-
Rosemary oil	17.4 ± 1^cd^	15.9 ± 0.5^ef^	9.7 ± 0^de^	-	23.4 ± 0.2^c^	21.9 ± 0.5^c^	14.5 ± 0.5^b^	8.3 ± 0.5^d^	17.5 ± 1^c^	15.1 ± 0.7^c^	9.1 ± 0.2^f^	-
Basil oil	8.9 ± 0.7^ghi^	-	-	-	8.23 ± 0.5^h^	-	-	-	10.5 ± 0.5^d^	8.2 ± 0.5^e^	-	-
Vetiver oil	11.5 ± 0.5^g^	10.3 ± 0.2	11.4 ± 0.5^de^	-	13.5 ± 0.5^ef^	11.7 ± 0.5^g^	8.2 ± 0.2^d^	-	-	-	-	-
Wintergreen	15.8 ± 0.7^ef^	14.8 ± 0.5 ^f^	9.5 ± 1 ^def^	-	10.2	-	-	-	8.9 ± 1^d^	-	-	-
Streptomycin**	18.4 ± 0.7				16.4 ± 0.2				21.2 ± 0.1			
Vehicle control	-				-				-			

** Streptomycin disc (25 μg) as a positive reference standard; Values are mean inhibition zone(mm) ± S.D of three replicates

Means within a column followed by different letters are statistically significant by tukey's test at p = 0.05; - no activity.

Minimum inhibitory concentration (MIC) for selected seven oils ranged from 0.8 to 12.8 mg/ml (Table 4). This study revealed that cinnamon oil showed maximum activity with MIC values ranging from 0.8 to 3.2 mg/ml followed by clove oil with MIC values ranging from 1.6 to 6.4 mg/ml against all the tested strains where as remaining oils showed moderate MIC values.

**Table 4:** Minimum Inhibitory Concentration (MIC) of selected essential oils (mg/ml).

**Oil name**	***S. aureus***	***B. subtilis***	***K. pneumoniae***	***P. vulgaris***	***P. aeruginosa***	***E. coli***
Cinnamon oil	3.2	>1.6	3.2	>1.6	>0.8	>1.6
Clove oil	>6.4	>3.2	>6.4	>3.2	>1.6	>1.6
Geranium oil	>12.8	>6.4	12.8	>12.8	>12.8	>6.4
Lemon oil	>12.8	>12.8	>12.8	>6.4	12.8	>6.4
Lime oil	12.8	>6.4	>6.4	>3.2	>6.4	>6.4
Orange oil	>12.8	>12.8	12.8	>6.4	>12.8	>12.8
Rosemary oil	>12.8	>6.4	>12.8	>6.4	>6.4	>6.4

GC/MS analysis of cinnamon oil identified thirty-eight phytochemicals as constituents; of these cinnamaldehyde was the major compound (52.4%) followed by Benzaldehyde (12.31%), benzoic acid (8.20%) and benzyl alcohol (2.23%). Remaining chemical compounds were in trace amounts. The major components and their retention times are summarized in Table 5.

**Table 5:** Major chemical compounds of cinnamon oil (GC-MS analysis)

**S. No**	**Rt (min)**	**Compound**	**Peak Area **[%]
1	7.51	Benzaldehyde	12.31
2	7.77	Benzyl alcohol	2.23
3	8.741	Cinnamaldehyde	52.42
4	9.88	Benzoic acid	8.20
5	-	Others	24.84

## Discussion

Plant essential oils and extracts have been used for many thousands of years [22], in food preservation, pharmaceuticals, alternative medicine and natural therapies [23,24]. It is necessary to investigate those plants scientifically which have been used in traditional medicine to improve the quality of healthcare. Essential oils are potential sources of novel antimicrobial compounds [25] especially against bacterial pathogens. *In vitro *studies in this work showed that the essential oils inhibited bacterial growth but their effectiveness varied. The antimicrobial activity of many essential oils has been previously reviewed and classified as strong, medium or weak [26].

In our study, cinnamon, clove, geranium, lemon, lime, orange and rosemary oils exhibited strong activity against the selected bacterial strains. Several studies [27,28] have shown that cinnamon, clove and rosemary oils had strong and consistent inhibitory effects against various pathogens. Even though earlier studies have reported better antimicrobial activity for eucalyptus oil [29,30] our study showed least inhibitory activity of eucalyptus in addition to aniseed and camphor oils. Among all oils analyzed in this work, the essential oil of cinnamon was the most effective as an antibacterial agent. The antibacterial activity has been attributed to the presence of some active constituents in the oils. Our GC-MS study revealed cinnamaldehyde to be the major constituent of cinnamon oil. Cinnamaldehyde was the predominant active compound found in cinnamon oil [31,32]. Earlier studies suggested that the antibacterial activity of cinnamon oil was probably due to their major component, cinnamaldehyde and their properties could be multiple. Cinnamaldehyde is a natural antioxidant and the animal studies suggest that an extract of cinnamon bark taken orally may help prevent stomach ulcer [33]. Cinnamaldehyde was completely inhibiting both sensitive and resistant stain of *Helicobacter pylori *[34]. Cinnamon oil was not harmful when consumed in food products and it inhibited the growth of molds, yeast and bacteria [27]. Cinnamon extract had a regulatory role in blood glucose level and lipids and it may also exert a blood glucose-suppressing effect [35]. The use of commercial cinnamon preparation produced an improvement of oral candidiasis of HIV-infected patients [36]. Cinnamon oil is locally applied with much benefit in neuralgia and headache. As an antiseptic it is used as an injection in gonorrhea; as germicide it is used internally in typhoid fever. This oil is also used in the treatment of cancer and other microbial diseases [37]. It can be incorporated into creams, lotions, drops, etc. which are applied externally on the body to treat diseases caused by *Aspergillus niger *[38].

An important characteristic of essential oils and their components is their hydrophobicity, which enable them to partition the lipids of the bacterial cell membrane and mitochondria, disturbing the cell structures and rendering them more permeable [39,40]. Extensive leakage from bacterial cells or the exit of critical molecules and ions will lead to death [41]. Gram-positive bacteria were more resistant to the essential oils than gram-negative bacteria [26]. In the present study, cinnamon, lime, geranium, rosemary, orange, lemon and clove oils were found to be equally effective against both gram-positive and gram-negative organisms.

## Conclusion

From this study it can be concluded that many essential oils possess antibacterial activity. Cinnamon oil has the most potential bactericidal properties. We believe that the present investigation together with previous studies provide support to the antibacterial properties of cinnamon oil. It can be used as antibacterial supplement in the developing countries towards the development of new therapeutic agents. Additional *in vivo *studies and clinical trials would be needed to justify and further evaluate the potential of this oil as an antibacterial agent in topical or oral applications.

## Competing interests

The author(s) declare that they have no competing interests.

## Authors' contributions

SP and MJ have carried out the experimental part such as selection of essential oils, inoculum preparation and antimicrobial evaluation. SI supervised the work, evaluated the results and corrected the manuscript for publication. All authors read and approved the final manuscript.

## Pre-publication history

The pre-publication history for this paper can be accessed here:


